# Spirulina Protects against Hepatic Inflammation in Aging: An Effect Related to the Modulation of the Gut Microbiota?

**DOI:** 10.3390/nu9060633

**Published:** 2017-06-20

**Authors:** Audrey M. Neyrinck, Bernard Taminiau, Hannah Walgrave, Georges Daube, Patrice D. Cani, Laure B. Bindels, Nathalie M. Delzenne

**Affiliations:** 1 Metabolism and Nutrition Research Group, Louvain Drug Research Institute, Université Catholique de Louvain, B-1200 Brussels, Belgium; audrey.neyrinck@uclouvain.be (A.M.N.); hannah.walgrave@student.kuleuven.be (H.W.); patrice.cani@uclouvain.be (P.D.C.); laure.bindels@uclouvain.be (L.B.B.); 2 Fundamental and Applied Research for Animal and Health (FARAH), Faculty of Veterinary Medicine, University of Liège, 4000 Liège, Belgium; bernard.taminiau@ulg.ac.be (B.T.); georges.daube@ulg.ac.be (G.D.); 3 Walloon Excellence in Life Sciences and BIOtechnology (WELBIO), Louvain Drug Research Institute, UCL, B-1200 Brussels, Belgium

**Keywords:** Spirulina, aging, microbiota, inflammaging, gut-liver axis

## Abstract

Aging predisposes to hepatic dysfunction and inflammation that can contribute to the development of non-alcoholic fatty liver disease. Spirulina, a cyanobacterium used as a food additive or food supplement, has been shown to impact immune function. We have tested the potential hepatoprotective effect of a Spirulina in aged mice and to determine whether these effects can be related to a modulation of the gut microbiota. Old mice have been fed a standard diet supplemented with or without 5% Spirulina for six weeks. Among several changes of gut microbiota composition, an increase in *Roseburia* and *Lactobacillus* proportions occurs upon Spirulina treatment. Interestingly, parameters related to the innate immunity are upregulated in the small intestine of Spirulina-treated mice. Furthermore, the supplementation with Spirulina reduces several hepatic inflammatory and oxidative stress markers that are upregulated in old mice versus young mice. We conclude that the oral administration of a Spirulina is able to modulate the gut microbiota and to activate the immune system in the gut, a mechanism that may be involved in the improvement of the hepatic inflammation in aged mice. Those data open the way to new therapeutic tools in the management of immune alterations in aging, based on gut microbe-host interactions.

## 1. Introduction

The human lifespan has been increased steadily during modern times, mainly due to medical advancements in various life-threatening diseases. However, this gratifying longevity rise is accompanied by growing incidences of devastating age-related pathologies [1]. Aging is a complex process defined as a progressive functional deterioration associated with frailty, disease, and death. Stress and immune responses deteriorate during aging, causing low-grade inflammation and increased susceptibility to infections, which collectively lead to severe diseases [2]. Specific age-related hepatic changes have been reported, such as an increased hepatocyte size, an increase in the number of binucleated cells, and a reduction in mitochondrial number [2]. These changes may significantly affect liver morphology, physiology, and oxidative capacity. In fact, the inflammaging theory can be applied to hepatic disorders: aging predisposes to hepatic functional and structural impairment, inflammation and metabolic risk, which favor non-alcoholic fatty liver disease (NAFLD) that can evolve into non-alcoholic steatohepatitis (NASH). Among liver diseases, evidence suggests that the prevalence of NASH and chronic liver disease is increased with age [3]. No validated treatments of NAFLD exist beyond weight loss or comorbidity management, thereby emphasizing the need to validate alternative dietary strategies that effectively mitigate the progression to NAFLD.

Due to adverse side effects commonly associated with drug therapy, public interest in natural products with health-promoting properties as an alternative to conventional drugs has rapidly grown [4]. Starting in the middle of the 1980s, significant efforts and extensive investigations have been devoted to the development of functional foods for preventing or managing various diseases.

Spirulina is a filamentous, spiral-shaped, water-based blue-green microalga [5,6]. Spirulina was initially classified in the plant kingdom because of its richness in plant pigments, as well as its ability of photosynthesis. It was later placed in the bacteria kingdom based on new understanding of its genetics, physiology, and biochemical properties [5]. Spirulina has been consumed as a food compound by North Africans and Mexicans because it contains high amounts of antioxidants, such as β-carotene, phycocyanin (containing an open-chain tetrapyrrole chromophore known as phycocyanobilin, which is covalently attached to the apoprotein), microelements (K, Na, Ca, Mg, Fe, Zn), vitamins (tocopherols), eight essential amino acids, polyunsaturated fatty acids, especially γ-linolenic acid, and phenolic compounds [6,7]. Moreover, the nutritional value of Spirulina is well recognized through its peculiar high protein content (60–70% of dry weight) [5]. Spirulina is often used as a dietary nutritional supplement in many countries due to its anti-inflammatory actions [6]. In fact, Spirulina is considered to be one of the most important healing and prophylactic nutritional ingredients of the 21st century due to its nutrient profile, its therapeutic effects, and the lack of toxicity [6]. Consumption of Spirulina as a dietary supplement is recommended in arterial hypertension, some inflammatory diseases, insulin-resistance, diabetes mellitus, non-alcoholic fatty liver disease, malnutrition, anemia, allergic rhinitis, cancer, and in reduction of drug toxicity [5,6,7].

Emerging evidence has revealed extensive crosstalk between the microbiota, the immune system, and inflammatory pathways that influence aging in humans. This interplay is affected by various genetic and environmental factors in addition to lifestyle. Recent studies shed light on the composition of the gut microbiota throughout life and explore their role in aging [1]. Indeed, with a global impact on the physiology of the intestinal tract, the aging process can seriously affect the composition of the human gut microbiota. The decreased intestinal motility results in a slower intestinal transit that affects defecation and leads to constipation. The subsequent reduced bacterial excretion alters the gut fermentative processes [8]. Inevitably, this affects the homeostasis of the bacterial ecosystem in the intestinal tract. Moreover, considering the impact of the diet on the gut microbiota composition, changes in nutritional behavior and lifestyle of the aged people concur to the age-related imbalance of the intestinal microbial community. Counteracting inflammaging occurring in the liver, among other sites, through gut microbiota modulation may be a key factor for healthy aging. The purpose of the present study was to determine whether the oral administration of Spirulina to aged mice could modulate gut microbiota composition and help to offset the inflammaging after six weeks of supplementation.

## 2. Materials and Methods

### 2.1. Animals and Diet Intervention

Male C57BL/6J mice of three and 24 months were purchased from Janvier Labs (Saint Berthevin, France) and maintained in a specific pathogen-free environment. Animals were housed in groups of three mice per cage in a controlled environment (12-h daylight cycle) with free access to food and water. Animal experiments were approved and performed in accordance with the guidelines of the local ethics committee. The ethical code is 2014/UCL/MD/022. Housing conditions were as specified by the Belgian Law of 29 May 2013, on the protection of laboratory animals (Agreement LA 1230314). After one week of acclimatization with a standard diet (AIN93M, Research Diet, New Brunswick, NJ, USA), mice were assigned into three groups: mice of three months were fed with the standard diet (young group) and mice of 24 months were fed a standard diet supplemented with or without 5% Spirulina (old-SP and old-CT groups, respectively) for six weeks. Biores (Liège, Belgium) supplied the Spirulina (*Arthrospira platensis*). The composition of the batch used for the study (expressed as dry weight) was 16% carbohydrates (including calcium-spirulan), 55% protein, 5–10% lipids, 4–8% fiber; the percentage of the most important pigment (phycocyanin) being 13.4% (expressed as dry weight). After six weeks of dietary treatment, 6 h-fasted mice were anaesthetized with isoflurane gas (Abbot, Ottignies, Belgium). Blood samples were harvested for further analysis. Mice were necropsied after cervical dislocation. Adipose tissues (epididymal, subcutaneous, visceral), liver, spleen, caecal content and tissue were weighted. Caecal content and tissue, liver, jejunum, ileum, and colon were collected, frozen in liquid nitrogen, and stored at −80 °C.

### 2.2. Gut Microbiota Analyses

Genomic DNA was extracted from the caecal content using a QIAamp DNA Stool Mini Kits (Qiagen, Hilden, Germany) according to the manufacturer’s instructions, including a bead-beating step. 16S rDNA profiling, targeting the V1–V3 hypervariable region and sequenced on Illumina MiSeq were performed as described previously [9] and detailed in Supplementary File 1. For total bacteria, *Roseburia* spp. and *Lactobacillus* spp. quantification by qPCR, primers, and conditions were based on the 16S rRNA gene sequence and was described earlier [10].

### 2.3. Biochemical Analysis

Triglycerides and cholesterol were measured in the liver tissue after extraction with chloroform–methanol according to the Folch method [11] and using kits coupling the enzymatic reaction and spectrophotometric detection of the final product (Diasys Diagnostic and System, Holzheim, Germany). Alanine aminotransferase (ALAT) levels were measured in the serum as a marker of liver damage using the ALAT/GPT kit (DiaSys Diagnostic and Systems). Plasma concentrations of cytokines (IL6, IL10, IL1β, MCP1, TNFα, and IFNγ) were determined using a multiplex immunoassay kit (Bio-Plex Cytokine Assay, Bio-Rad, Nazareth, Belgium).

### 2.4. Tissue mRNA Analyses

Total RNA was isolated from tissues using the TriPure isolation reagent kit (Roche Diagnostics, Penzberg, Germany). Complementary DNA was prepared by reverse transcription of 1 µg of total RNA using the Kit Reverse Transcription System (Promega, Madison, WI, USA). Real-time polymerase chain reaction (PCR) was performed with a StepOnePlus Real-Time PCR System and software (Applied Biosystems, Den Ijssel, The Netherlands) using SYBR Green (Eurogentec, Seraing, Belgium) for detection. All samples were run in duplicate in a single 96-well reaction plate, and data were analyzed according to the 2^−∆∆CT^ method. The purity of the amplified product was checked by analyzing the melting curve performed at the end of the amplification step. The ribosomal protein L19 (RPL19) gene was chosen as the house-keeping gene. Primer sequences are given in Supplementary Table S1.

### 2.5. TLR2 and TLR4 Agonists

Toll like receptor (TLR) 2 and TLR4 agonists were measured using Hek-Blue reporter cell lines according to manufacturer instructions (InvivoGen, San Diego, CA, USA). Cells were maintained in RPMI medium with ultra-low endotoxin FBS (Biosera, Nuaille, France) with appropriate antibiotics (Normocyn, Zeocyn and/or HekBlue selection, InvivoGen, San Diego, CA, USA). We resuspended fecal material or Spirulina in LAL reagent water (Lonza, Walkersville, MD, USA) to a final concentration of 100 mg/mL and homogenized for 4 min using a tissue lyzer without the addition of beads to avoid bacteria disruption. We then centrifuged the samples at 8000× *g* for 2 min, serially diluted the resulting supernatant, heat them at 56 °C for 45 min, and applied them to cells. Purified *Escherichia coli* LPS (Sigma, St. Louis, MO, USA) and FSL-1 (InvivoGen, San Diego, CA, USA) were used for standard curve determination using HEK-Blue mTLR4 and HEK-Blue mTLR2 cells, respectively. After 21 h of stimulation, we applied cell culture supernatant to QUANTI-Blue medium (InvivoGen, San Diego, CA, USA) and measured the alkaline phosphatase activity at 620 nm after 3 h. A control cell line (HEK-Blue Null1 cells) was included to remove unspecific signals from the fecal slurry.

### 2.6. Statistical Analysis

Results are presented as means with their standard errors, if not indicated otherwise. Statistical analysis was performed by one-way analysis of variance (ANOVA) followed by post hoc Tukey’s multiple comparison tests using GraphPad Prism software (version 5.00, GraphPad Software, San Diego, CA, USA). The results were considered statistically significant at *p* < 0.05.

## 3. Results

### 3.1. Spirulina Did Not Change Body Weight Gain, Food Intake, and Organ Weights of Old Mice

The body weight gain was much lower in old mice than in young mice. Food intake, adiposity, spleen weight, and caecum weight were not significantly different in aged mice versus young mice (Table 1). However, we observed higher liver weight in aged mice as compared to young mice. Spirulina supplementation did not influence significantly any of these parameters.

### 3.2. Spirulina Changed Microbial Diversity and Populations in Old Mice

**Figure 1:**
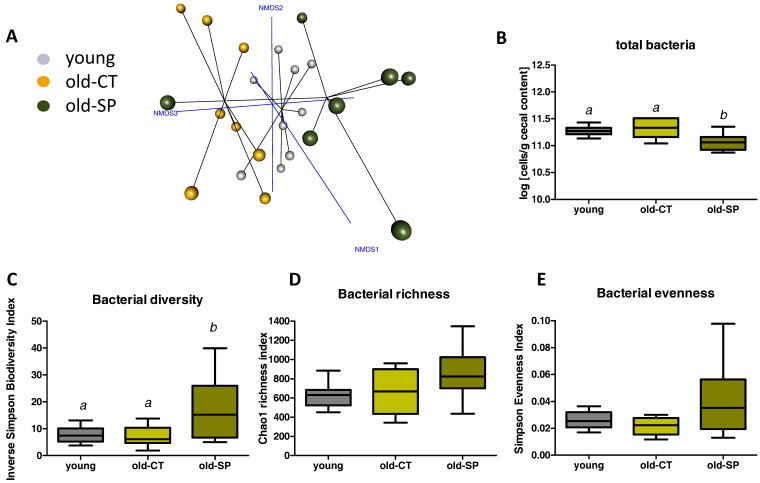
Non-metric dimensional scaling built on a Bray-Curtis distance matrix computed at the species level (**A**); Total bacteria measured by qPCR (**B**); Bacterial diversity deduced from the inverse Simpson Index (**C**); Bacterial richness deduced from the Chao1 Index (**D**); Bacterial evenness deduced from the Simpson Index (**E**). Old mice were fed a standard diet supplemented with or without Spirulina for six weeks and were compared to young mice fed a standard diet. Data of the whiskers plots show the minimum and maximum. Data with different superscript letters are significantly different at *p* < 0.05 (ANOVA).

Differences between the groups in terms of intestinal microbial population structure were visualized by non-metric dimensional scaling built upon a Bray-Curtis distance matrix computed at the species level (Figure 1A). A distinct cluster was observed for each of the three groups of mice and was confirmed by permutational AMOVA analysis of the microbial population structure (*F* score = 5.369, *p* = 0.001 old-CT versus young groups, and *F* score = 2.392, *p* = 0.006 old-SP versus old-CT groups). Spirulina supplementation decreased the number of bacteria in the caecal content of old mice and changed caecal microbial diversity, likely due to bacterial evenness (Figure 1B–E).

**Figure 2:**
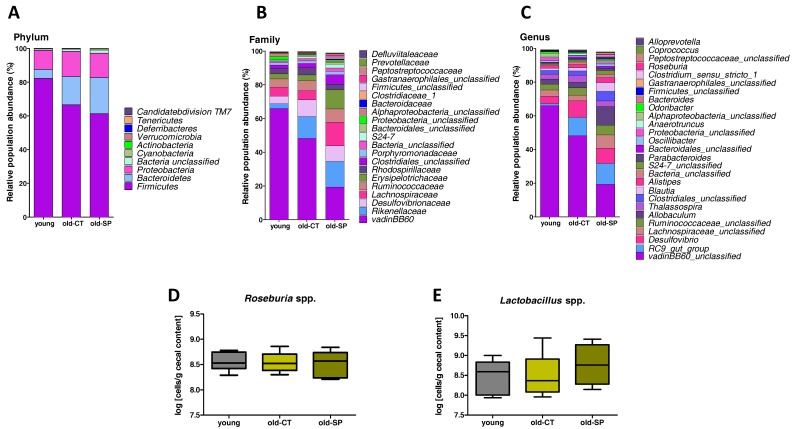
Changes in microbial populations in the caecal content, assessed by 16S profiling (**A**–**C**) and qPCR (**D**–**F**). Relative abundances of bacterial phyla (**A**) and bacterial taxa accounting for more than 1%, at the family (**B**) and genus levels (**C**). qPCR analysis of *Roseburia* spp. (**D**) and *Lactobacillus* spp. (**E**) in the caecal content. Old mice were fed a standard diet supplemented with or without Spirulina for six weeks and were compared to young mice fed a standard diet.

To assess specific changes in intestinal microbiota, we compared the relative abundance of bacterial taxa between treatment groups (Figure 2A–C and Supplementary Table S2). Although bacterial diversity, bacterial evenness, and bacterial richness were not significantly different between the old group and the young group, aging induced a significant phylum-wide shift from Firmicutes to Bacteroidetes, which was not modified by the Spirulina supplementation. At the family level, the abundance of the vadinBB60 group decreased, whereas proportions of the *Desulfovibrionaceae* and *Rikenellaceae* increased in old mice as compared to young mice. Those effects were not counteracted by the Spirulina treatment. However, we observed a higher proportion of the *Lachnospiraceae* in the old-SP group. The abundance of the vadinBB60 group was the lowest in the old-SP group as compared to the young group and the old-CT group. At a lower taxonomic level, the most prominent differences observed upon Spirulina supplementation related to the *Roseburia* and *Lactobacillus* genera, whose relative abundance increased by 12- and 15-fold in Spirulina-fed mice, respectively. To a lesser extent, the proportion of *Blautia* was also increased upon Spirulina treatment. However, we did not observe any changes in *Roseburia* spp. and *Lactobacillus* spp. in the caecal content by qPCR analysis (Figure 2D,E).

### 3.3. Spirulina Upregulated the Expression of Antimicrobial Peptides (AMPs) in the Small Intestine of Old Mice

**Figure 3:**
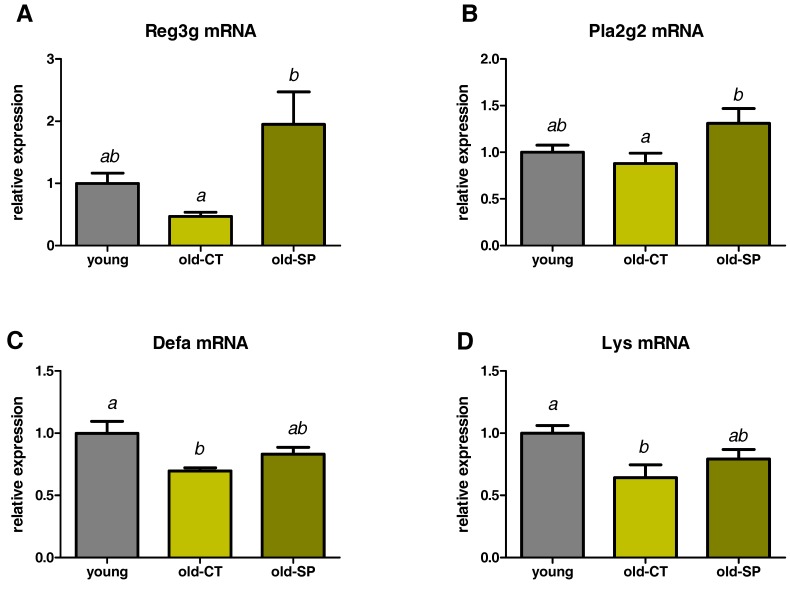
Effect of Spirulina on the expression of antimicrobial peptides in the ileum. RegIIIγ encoded by Reg3g (**A**), phospholipase A2 group II encoded by Pla2g2 (**B**), α-defensins encoded by Defa (**C**), and lysozyme C encoded by Lys (**D**). Old mice were fed a standard diet supplemented with or without Spirulina for six weeks and were compared to young mice fed a standard diet. Data with different superscript letters are significantly different at *p* < 0.05 (ANOVA).

Multiple host factors are likely to contribute to changes in the intestinal microbiome during aging, and one of them is the intestinal innate immune system. Antimicrobial molecules, which are part of the innate immune response, are secreted from epithelial cells or Paneth cells and contribute to shaping the composition of the gut microbiota [12]. These peptides are an attractive mechanism to explain how nutrients can modulate the gut ecosystem [13]. We measured the expression of secreted AMP produced by Paneth cells and/or enterocytes in the ileum: C-type lectin, primarily the regenerating islet-derived 3-gamma (RegIIIγ), phospholipase A2g2 (Pla2g2), α-defensins (Defa), and lysozyme C (Lys). AMP expressions were downregulated in old mice; those effects being significant for α-defensins and lysozyme C (Figure 3). Interestingly, Spirulina significantly increased the expression of Pla2g2 and RegIIIγ, suggesting that Spirulina improved innate immunity through the secretion of AMPs.

### 3.4. Spirulina Upregulated Parameters Related to the Innate Immunity in the Small Intestine of Old Mice

**Figure 4:**
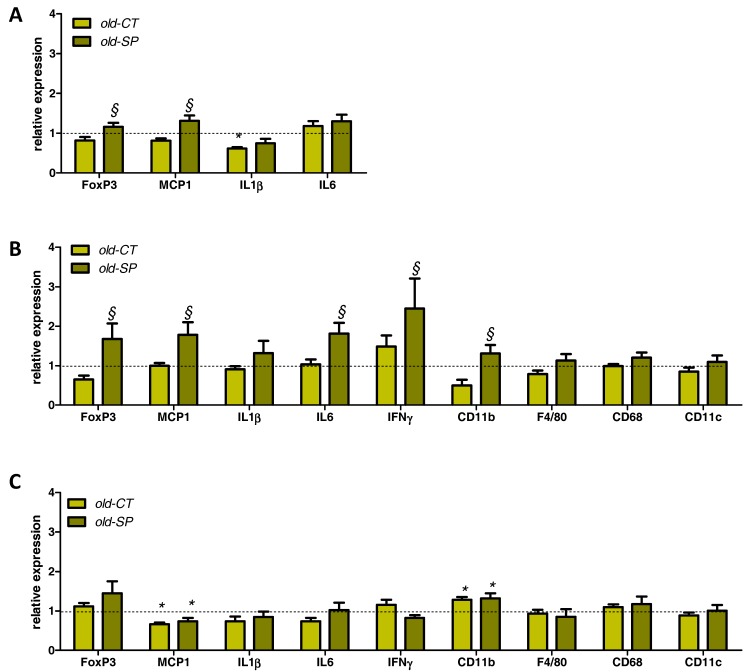
Expression of inflammatory markers in the jejunum (**A**), ileum (**B**), and colon (**C**). Old mice were fed a standard diet supplemented with or without Spirulina for six weeks and were compared to young mice fed a standard diet. Values are expressed as relative units with the mean of young mice values set at 1. * *p* < 0.05 versus young mice; ^§^ *p* < 0.05 versus old-CT mice (ANOVA). IFNγ, interferon gamma; IL, interleukin; MCP1, monocyte chemotactic protein-1.

**Figure 5:**
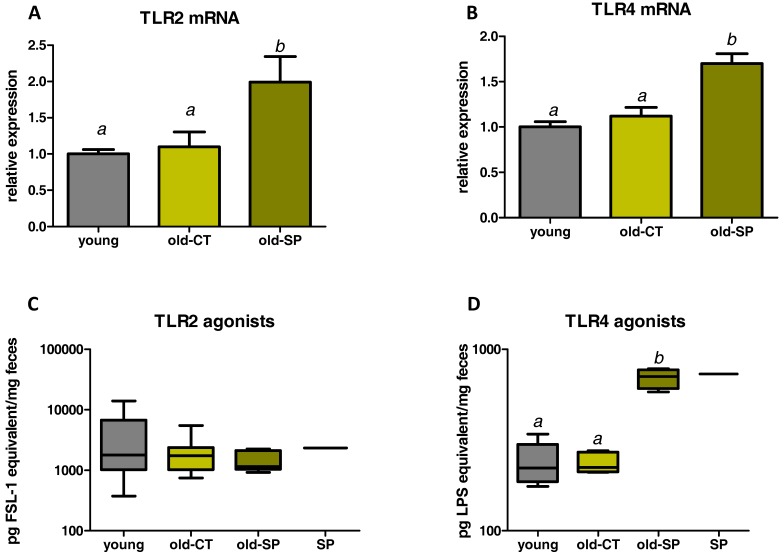
Expression of TLR2 (**A**) and TLR4 (**B**) in the ileum. Levels of TLR2 (**C**) and TLR4 (**D**) agonist activities in feces and in Spirulina, itself. Old mice were fed a standard diet supplemented with or without Spirulina for six weeks and were compared to young mice fed a standard diet. Data with different superscript letters are significantly different at *p* < 0.05 (ANOVA). TLR, toll like receptor.

We analyzed the expression of some markers related to the immune system in the jejunum (Figure 4A). IL1β was downregulated in aged mice but mRNA level was not restored by the Spirulina supplementation. Conversely, expression of the transcription factor Foxp3 (involved in the differentiation of T cells into regulatory T cells (Tregs)) and of the monocyte chemotactic protein-1 (MCP1, a chemoattractant cytokine) were increased due to Spirulina treatment in old mice. A panel of markers involved in gut immune function were also analyzed in the ileum and in the colon (Figure 4B,C). Old mice that consumed Spirulina exhibited activation of several immune parameters in the ileum—in particular, Foxp3—suggesting an improvement of the gut immune function upon Spirulina treatment in this segment (Figure 4B). Of note, the expression of IL10 in the ileum was not affected by the treatment (data not shown). In contrast to what happens in the jejunum or ileum, Spirulina supplementation had no effect on immune/inflammatory parameters in the colon. Only CD11b was upregulated in the colon of old mice versus the young mice, whereas we observed a decrease in MCP1 mRNA content (Figure 4C). We analyzed TLR pathways by measuring both TLR2 and TLR4 expression in the ileum and TLR2/TLR4 agonist activities using Hek-Blue reporter cell lines (Figure 5). Spirulina supplementation upregulated both TLR2 and TLR4 expression in the ileum of aged mice (Figure 5A,B). No difference in faecal TLR2 agonists were found between groups, whereas the TLR4 agonist activity was increased by three-fold in fecal material from old-SP mice. In accordance with these results, a solution of Spirulina (5%) exhibited a TLR4 agonist activity similar to the one reached in old-SP mice, suggesting a direct effect of the Spirulina, itself, on the TLR4 pathway.

### 3.5. Spirulina Reduced Several Hepatic Inflammatory and Oxidative Stress Markers in Old Mice without Affecting Lipid Content

Hepatic contents in lipids (triglycerides and cholesterol) and serum alanine aminotransferase (ALAT), measured as a marker of liver damage, were not significantly modified with aging (Table 2). Furthermore, Spirulina treatment had no significant impact on these parameters when compared with the old-CT group.

**Figure 6:**
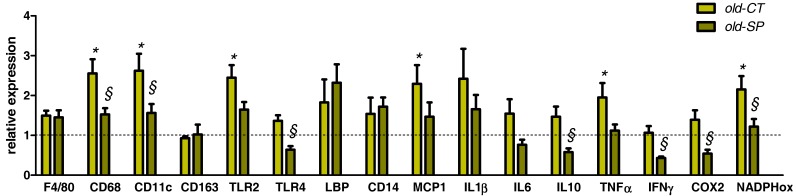
Expression of inflammatory and oxidative markers in the liver. Old mice were fed a standard diet supplemented with or without Spirulina for six weeks and were compared to young mice fed a standard diet. Values are expressed as relative units with the mean of young mice values set at 1; * *p* < 0.05 versus young mice; ^§^ *p* < 0.05 versus old-CT mice (ANOVA). COX2, cyclooxygenase 2; IFNγ, interferon gamma; IL, interleukin; LBP, lipopolysaccharide binding protein; MCP1, monocyte chemotactic protein-1; NADPHox, NADPH oxidase; TLR, toll like receptor; TNFα, tumor necrosis factor alpha.

Aged mice exhibited inflammation and oxidative stress in the liver tissue as compared to young mice (Figure 6). Interestingly, several parameters involved in inflammatory (CD68, CD11c, TLR4, IL10, IFNγ, COX2) or oxidative (NADPHoxidase) processes were significantly downregulated upon Spirulina treatment.

Of note, analysis of inflammatory parameters in the plasma from vena cava did not reveal any significant effect of aging or Spirulina supplementation (Supplementary Figure S1).

## 4. Discussion

The gut microbiota is required for metabolic and immune homeostasis in adult life. Compositional changes of gut microbiota have been linked with inflammatory and metabolic disorders occurring in inflammatory bowel disease, irritable bowel syndrome, and obesity in humans [14]. The maintenance of the gut microbial ecosystem could be essential for the preservation of key gut functions during the aging process [14]. In this perspective, dietary manipulation of the gut microbiota of the elderly may represent a tool for preserving a healthy gastrointestinal microbial community. Bacteroidetes and Firmicutes are the most dominant phyla in the gut. The ratio between these two phyla of bacteria has been proposed as an informative parameter of the overall status of the gut microbiota, but this clearly remains a matter of debate [15]. In the present study, the Firmicutes/Bacteroidetes ratio decreased in old mice as compared to young mice. Several studies reported that the Firmicutes/Bacteroidetes ratio was lower in elderly people than in young adults [16]. Furthermore, we observed a higher proportion of bacteria related to endotoxin-producing opportunistic pathogens, such as *Desulfovibrionaceae* in old mice [17]. This result supports the available studies reporting an age-related increase in facultative anaerobes which can overgrow in certain situations, such as inflammation. These changes in gut microbiota can be the cause of infections when the host resistance mechanisms fail as a result of the aging process [16]. Although *Desulfovibrionaceae* were not significantly modified by the Spirulina supplementation, we reported interesting changes in gut microbiota composition in old mice due to Spirulina supplementation. Among the bacteria potentially beneficial for host physiology, the relative abundance of *Roseburia* and *Lactobacillus* increased upon Spirulina treatment in old mice. The increase in the relative abundance of these bacteria is likely due to a lack of change in the absolute numbers of these bacteria per gram of caecal content in combination with a decrease in the number of total bacteria per gram of caecal content. This suggests that the antimicrobial activity of Spirulina targets other bacteria (perhaps bacteria belonging to the vadinBB60 group since their proportion decreased). Different studies have shown that the proportions of *Roseburia* and *Lactobacillus* were significantly decreased in mice fed an obesogenic high-fat diet, whereas treatments which prevent high fat diet-induced inflammatory disorders increased the abundance of those genera [18]. There are three major groups of H_2_-consuming microorganisms (hydrogenotrophs) in the human colon: methanogens, sulfate-reducing bacteria (such as bacteria from *Desulfovibrionaceae)*, and acetogens (such as bacteria from *Lachnospiraceae*). Direct competition among them may occur for the common substrate H_2_ [19]. In particular, inside the Firmicutes phylum, the higher abundance of acetogenic *Blautia* (from the *Lachnospiraceae* family) observed with Spirulina supplementation may determine the biomass specific growth rate of the other hydrogenotrophs associated with altered metabolism or inflammation in the host [20,21,22,23].

By which mechanisms would Spirulina intake change the composition of the intestinal microbiota? One of the mechanisms could be the presence of antimicrobial substances produced by Spirulina [24]. For example, antibacterial properties have been demonstrated for phycocyanin from Spirulina platensis against *Escherichia coli*, *Klebsiella pneumoniae*, *Pseudomonas aeruginosa*, and *Staphylococcus aureus* [25]. On the other hand, antimicrobial peptides (AMPs) could be mediators of the nutritional modulation of the gut microbiota. In the present study, RegIIIγ and Pla2g2 were increased by the supplementation with Spirulina, suggesting that the host contributes to the reduction and modification of the microbial community by modulating the production of specific AMPs [9,13].

Modulation of the gut microbiota is one of the important factors influencing gut immunity [26]. The interaction between microbial components and TLRs—which are innate immune sensors present on certain immune cells within the gut—contribute to the maintenance of the mucosal and systemic immune status [27]. Indeed, TLRs may be considered an interface between microbiota and the intestinal epithelial barrier and immune system. TLR ligands stimulate TLR expressing cells, such as macrophages, to produce proinflammatory cytokines including IL1β and chemokines such as MCP1, which recruits inflammatory macrophages [27]. We showed that TLR4 agonist activity was increased in fecal samples of old-SP mice to a similar level that the one conferred by the extract alone. These data pinpoint the likelihood of a direct effect of Spirulina independently of gut bacteria. Consequently we observed upregulation of TLR4 expression in the small intestine of old mice treated with Spirulina. Although fecal samples from old-SP mice did not exhibit TLR2 agonist activities, the expression of TLR2 was upregulated in the ileum of Spirulina-treated mice. This increase in TLR2 expression is, therefore, achieved independently of a TLR2 direct stimulation and could be related to an increased presence of dendritic cells (CD11b) in the ileum. Indeed, our results demonstrated that supplementation with Spirulina increased the expression of CD11b and Foxp3 in the small intestine, suggesting a higher number of dendritic cells and regulatory T cells in this gut segment, respectively. It has been shown that a specific subset of dendritic cells characterized by the CD11b marker is able to secrete high levels of IL6 and direct the differentiation of T cells in mice [26,28]. Therefore, we can postulate that the Spirulina may increase the numbers of CD11b^+^ dendritic cells of aged mice and to stimulate them both to produce higher levels of IL6 and to differentiate T cells into Tregs that have also an essential role in maintaining immune tolerance in the gut. Such results have already been reported with a high molecular weight polysaccharide fraction from Spirulina [26]. Altogether, those data indicate an improvement of the gut immune function of old mice after oral intake of Spirulina. This could be determinant in the decline of regulatory immune function at the gut mucosa level occurring upon aging [29]. However, we also observe an increase in proinflammatory markers (MCP1, IL6, IFNγ) in the intestinal tissue together with the TLR4 upregulation. This immunostimulatory effect can be seen as a double-edged sword. Immunostimulation can be seen as beneficial in some contexts such as healthy ageing while being potentially detrimental in specific inflammatory settings, such as inflammatory bowel disease.

Intestinal mucosal barrier dysfunction is closely related to liver diseases, which implies an impaired gut-liver axis. Aging does not promote the development of hepatic steatosis, but leads to increased hepatocellular injury and inflammation that may be due, in part, to increased M1 macrophage polarization [3]. In our study, we observed that old mice exhibited inflammaging (highlighted significantly by the higher expression of TNFα and MCP1), and oxidative stress (as shown by the NADPH oxidase expression) in the liver tissue as compared to young mice, whereas hepatic content of lipids was unchanged. Macrophage activation and M1 macrophage polarization were evidenced by the higher expression of CD68 and CD11c, respectively, whereas the expression of a marker related to hepatic M2 macrophages (CD163) or to mature macrophages (F4/80) were unaffected by age. Interestingly, Spirulina blunted hepatic inflammation induced in aging. Indeed, our data showed that Spirulina administration significantly suppressed the expression of TNFα, COX-2, IFNγ, and MCP1 in old mice. Moreover, macrophage activation in the liver was decreased by the Spirulina, as shown by the significant downregulation of CD68 and CD11c. Importantly, in contrast to what happened in the gut, supplementation with Spirulina was found to also inhibit the expression of TLR4 in the liver. In fact, alteration of TLR signaling is one of the major mechanisms involved in the impairment of immune response because TLR serve as pattern recognition receptors on macrophages and other innate immune cell types [30]. Consequently, we would suggest an inhibition of the TLR4-dependent inflammation in liver tissue after Spirulina supplementation, which would reflect an enhancement of the hepatic immune response. Such a result has been already described in a rat model of NASH in which Spirulina administration inhibited NASH progression through antioxidative and anti-inflammatory mechanisms [31]. Given that the liver and gut are connected through the portal vein, we can speculate that anti-inflammatory effects of Spirulina in the liver may be dependent on improvements of the gut immune system or changes in gut barrier function via the production of AMPs [9,13]. Anti-inflammatory effects of phycocyanin present in the Spirulina (or its chromophore phycocyanobilin) could be another mechanism explaining the anti-inflammatory effect in the liver, independently of a modulation of the gut microbiota and/or the gut immune function. Indeed, phycocyanin has been shown to decrease Kupffer cell phagocytosis and the associated respiratory burst activity in vivo, effects that may contribute to the abolition of inflammatory and oxidative stresses [32]. Another study revealed the inhibitory effect of C-phycocyanin on the TNF-α response induced by lipopolysaccharide in mice, a response known to depend mainly upon Kupffer cell function, thus supporting the anti-inflammatory potential of the pigment [33]. Finally, it has been demonstrated that phycocyanin administration in a rat model of NASH might lessen the inflammatory response through antioxidative and anti-inflammatory mechanisms, and effectively inhibit NASH progression [31].

## 5. Conclusions

In conclusion, the results from the present study indicate that oral feeding of a Spirulina modulates several immunological functions involving, among others, the TLR4 pathway in old mice. The fact that its oral consumption can influence both gut immunity and systemic sites, such as the liver, suggests that its immune action is not confined to the gut immune system. Moreover, improvement of the homeostasis in the gut ecosystem could be essential for the gut health during the aging process, and, in this perspective, dietary manipulation of the gut microbiota of the elderly with Spirulina, may represent a tool for preserving a healthy gastrointestinal microbial community in addition to its beneficial effects on immune function.

## Figures and Tables

**Table 1:** Body weight gain, food intake, and organ weights.

	Young	Old-CT	Old-SP
Body weight gain (g)	3.11 ± 0.38 ^a^	0.23 ± 0.29 ^b^	0.64 ± 0.65 ^b^
Total food intake (g)	108 ± 7	130 ± 17	121 ± 2
Visceral adipose tissue (g)	0.24 ± 0.03	0.25 ± 0.03	0.19 ± 0.04
Subcutaneous adipose tissue (g)	0.42 ± 0.04	0.32 ± 0.04	0.30 ± 0.05
Epididymal adipose tissue (g)	0.51 ± 0.05	0.37 ± 0.05	0.33 ± 0.08
Liver (g/100 g body weight)	3.71 ± 0.09 ^a^	4.25 ± 0.24 ^b^	4.09 ± 0.10 ^a,b^
Spleen (g/100 g body weight)	0.30 ± 0.01	0.41± 0.06	0.35 ± 0.06
Caecal tissue (g/100 g body weight)	0.64 ± 0.06	0.62 ± 0.03	0.61 ± 0.06
Caecal content (g)	0.17 ± 0.02	0.19 ± 0.01	0.19 ± 0.02

Old mice were fed a standard diet supplemented with or without Spirulina for six weeks and were compared to young mice fed a standard diet. Data with different superscript letters are significantly different at *p* < 0.05 (ANOVA).

**Table 2:** Parameters related to liver lipid accumulation and hepatotoxicity.

	Young	Old-CT	Old-SP
Triglyceride content (nmol/mg tissue)	15.1 ± 1.7	10.4 ± 0.7	13.8 ± 2.2
Cholesterol content (nmol/mg tissue)	7.4 ± 0.6 ^a^	9.3 ± 0.3 ^a,b^	10.8 ± 1.3 ^b^
ALAT (U/L)	4.1 ± 1.2	3.1 ± 0.3	3.8 ± 0.8

Old mice were fed a standard diet supplemented with or without Spirulina for six weeks and were compared to young mice fed a standard diet. Data with different superscript letters are significantly different at *p* < 0.05 (ANOVA). ALAT, alanine aminotransferase.

## Supplementary Materials

The following are available online at www.mdpi.com/2072-6643/9/6/633/s1, Figure S1: Inflammatory markers measured in the plasma using Luminex^®^ technology, Table S1: Primer sequences used for quantitative PCR, Table S2: Abundance of bacteria taxa, expressed in percentage, that are statistically impacted by the dietary treatment as determined by pyrosequencing of 16sRNA gene, File S1: 16S rDNA high-throughput sequencing.
