# Supplementary material for: Spirulina Protects against Hepatic Inflammation in Aging: An Effect Related to the Modulation of the Gut Microbiota?
Source: Nutrients. 2017 Jun 20;9(6):633. doi: 10.3390/nu9060633 (PMC5490612; doi:10.3390/nu9060633)
Supplement: Supplementary file 1 [file nutrients-09-00633-s001.zip › nutrients-199449-supplementary.pdf]

Supplementary File S1. 16S rDNA high throughput sequencing

PCR-amplification of the V1–V3 region of the 16S rDNA and library preparation were performed with the following primers (with Illumina overhand adapters): forward (5′-TCGTCGGCAGCGTCAGATGTGTATAAGAGACAG-3′) and reverse (5′-GTVTVGTGGGCTCGGAGATGTGTATAAGAGACAG-3′). Each PCR product was purified with the Agencourt AMPure XP bead kit (Beckman Coulter, Pasadena, CA, USA) and submitted to a second PCR round for indexing, using the Nextera XT index primers 1 and 2. After purification, PCR products were quantified using the Quant-IT PicoGreen (ThermoFisher Scientific, Waltham, MA, USA) and diluted to 10 ng·μL^{-1}. A final quantification, by qPCR, of each sample in the library was performed using the KAPA SYBR® FAST qPCR Kit (KapaBiosystems, Wilmington, MA, USA) before normalization, pooling and sequencing on a MiSeq sequencer using v3 reagents (ILLUMINA, San Diego, CA, USA).

Sequence reads processing was performed as previously described using, respectively, MOTHUR software package v1.35 [1,2] and the Pyronoise algorithm and UCHIME algorithm [3], for alignment and clustering, denoising, and chimera detection. 16S reference alignment and taxonomical assignation were based upon the SILVA database (v1.19) of full-length 16S rDNA sequences[4].

Subsample datasets were obtained and used to evaluate the ecological indicators, richness estimation (Chao1 estimator), microbial biodiversity (reciprocal Simpson index), and the population evenness (derived from Simpson index) using MOTHUR [5]. The population structure and community membership were assessed with MOTHUR using distance matrices based on the Bray-Curtis dissimilarity index (a measure of community structure which considers shared OTUs and their relative abundances).

Ordination analysis and 3D plots were performed with Vegan, Vegan3d, and rgl packages in R (https://CRAN.R-project.org/package=vegan; https://CRAN.R-project.org/package=vegan3d; https://CRAN.R-project.org/package=rgl). Non-metric dimensional scaling, based upon the Bray-Curtis dissimilarity matrix, was applied to visualize the biodiversity between the groups. An AMOVA test was performed to assess the diversity clustering of treatment groups with the Bray-Curtis matrix using MOTHUR [6]. Statistical differences between bacterial biodiversity, richness, and evenness were assessed with two-way ANOVA corrected for multi-testing (Benjamini-Hochberg using PRISM 6 (Graphpad Software, La Jolla, CA, USA)), and differences were considered significant for a *p*-value of less than 0.05. Statistical difference of population abundance between treatment groups were assessed with ANOVA, corrected for mutli-testing (Benjamini-Hochberg False Discovery Rate) using STAMP software [7]. Statistical paired differences between treatment groups of specific bacterial populations were assessed by two-way ANOVA and the Tukey-Kramer post-hoc test using PRISM 6 (Graphpad Software, La Jolla, CA, USA), and differences were considered significant for a *p*-value of less than 0.05. All of the biosample raw reads have been deposited at the National Center for Biotechnology Information (NCBI) and are available under the Bioproject ID PRJNA348805.

1. Schloss, P.D.; Westcott, S.L.; Ryabin, T.; Hall, J.R.; Hartmann, M.; Hollister, E.B.; Lesniewski, R.A.; Oakley, B.B.; Parks, D.H.; Robinson, C.J., *et al.* Introducing mothur: Open-source, platform-independent, community-supported software for describing and comparing microbial communities. *Appl.Environ.Microbiol.* **2009**, *75*, 7537-7541.
2. Neyrinck, A.M.; Etxeberria, U.; Taminiau, B.; Daube, G.; Van Hul, M.; Everard, A.; Cani, P.D.; Bindels, L.B.; Delzenne, N.M. Rhubarb extract prevents hepatic inflammation induced by acute alcohol intake, an effect related to the modulation of the gut microbiota. *Mol Nutr Food Res* **2016**.
3. Edgar, R.C.; Haas, B.J.; Clemente, J.C.; Quince, C.; Knight, R. Uchime improves sensitivity and speed of chimera detection. *Bioinform.* **2011**, *27*, 2194-2200.
4. Quast, C.; Pruesse, E.; Yilmaz, P.; Gerken, J.; Schweer, T.; Yarza, P.; Peplies, J.; Glockner, F.O. The silva ribosomal rna gene database project: Improved data processing and web-based tools. *Nucleic Acids Res* **2013**, *41*, D590-596.
5. Hunter, P.R.; Gaston, M.A. Numerical index of the discriminatory ability of typing systems: An application of simpson's index of diversity. *J.Clin.Microbiol.* **1988**, *26*, 2465-2466.
6. Martin, A.P. Phylogenetic approaches for describing and comparing the diversity of microbial communities. *Appl.Environ.Microbiol.* **2002**, *68*, 3673-3682.
7. Parks, D.H.; Beiko, R.G. Identifying biologically relevant differences between metagenomic communities. *Bioinformatics.* **2010**, *26*, 715-721.

Supplementary Figure S1. Inflammatory markers measured in the plasma using Luminex® technology.

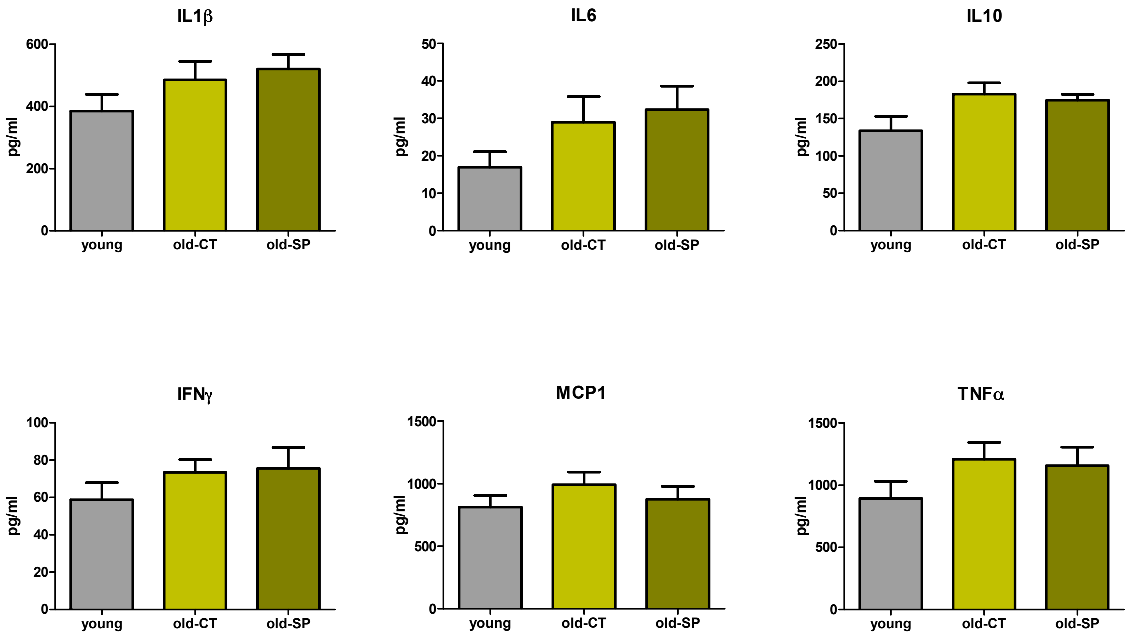

Old mice were fed a standard diet supplemented with or without Spirulina for six weeks and were compared to young mice fed a standard diet. IFNγ, interferon gamma; IL, interleukin; MCP1, monocyte chemotactic protein-1; TNFα, tumor necrosis factor alpha.

Supplementary Table S1. Primer sequences used for quantitative PCR.

	Primer Forward	Primer Reverse
RPL19	GAAGGTCAAAGGGAATGTGTTCA	CCTTGTCTGCCTTCAGCTTGT
Reg3γ	TTCCTGTCCTCCATGATCAAA	CATCCACCTCTGTTGGGTTC
Pla2g2	AAGGATCCCCCAAGGATGCCAC	CAGCCGTTTCTGACAGGAGTTCTGG
Defa	GGTGATCATCAGACCCCAGCATCAGT	AAGAGACTAAAACTGAGGAGCAGC
Lys	GCCAAGGTCTACAATCGTTGTGAGTTG	CAGTCAGCCAGCTTGACACCACG
FoxP3	TCCTTCCCAGAGTTCTTCCA	CGAACATGCGAGTAAACCAA
MCP1	GCAGTTAACGCCCCACTCA	CCCAGCCTACTCATTGGGATCA
IL1β	TCGCTCAGGGTCACAAGAAA	CATCAGAGGCAAGGAGGAAAAC
IL6	ACAAGTCGGAGGCTTAATTACACAT	TTGCCATTGCACAACTCTTTTC
IFNγ	TTCTTCAGCAACAGCAAGGC	ACTCCTTTTCCGCTTCCTGA
CD11b	GTCAGAGTCTGCCTCCGTGT	CCTGCGTGTGTTGTTCTTTG
F4/80	TGACAACCAGACGGCTTGTG	CAGGCGAGGAAAAGATAGTGT
CD68	CTTCCCACAGGCAGCACAG	AATGATGAGAGGCAGCAAGAGG
CD11c	ACGTCAGTACAAGGAGATGTTGGA	ATCCTATTGCAGAATGCTTCTTTACC
TLR4	CCCTCAGCACTCTTGATTGC	TGCTTCTGTTCCTTGACCCA
TLR2	CACCACTGCCCGTAGATGAA	GCCTCGGAATGCCAGCTT
CD163	GGCAACAAATACGTGGCTCT	ATGGGATTTCTCCTCCAACC
LBP	GTCCTGGGAATCTGTCCTTG	CCGGTAACCTTGCTGTTGTT
CD14	CCTGCCCTCTCCACCTTAGAC	TCAGTCCTCTCTCGCCCAAT
IL10	GGACAACATACTGCTAACCGAC	AAAATCACTCTTCACCTGCTCG
TNFα	AGCCCCCAGTCTGTATCCTT	GGTCACTGTCCCAGCATCTT
COX2	TGACCCCCAAGGCTCAAATAT	TGAACCCAGGTCCTCGCTTA
NADPHox	TTGGGTCAGCACTGGCTCTG	TGGCGGTGTGCAGTGCTATC

Supplementary Table S2. Abundance of bacteria taxa expressed in percentage, that are statistically impacted by the dietary treatment as determined by pyrosequencing of 16sRNA gene.

	Corrected *p*-value	Young mean	Young SD	Old-CT mean	Old-CT SD	Old-SP mean	Old-SP SD
**At the phylum level**							
Firmicutes	7.57×10^{-6}	82.257[a]	4.981	66.581[b]	19.949	61.287[b]	13.656
Bacteroidetes	7.58×10^{-6}	5.284[a]	2.612	16.846[b]	10.530	21.511[b]	11.672
**At the family level**							
Desulfovibrionaceae	1.09×10^{-2}	4.493[a]	1.707	9.946[b]	5.903	9.122[b]	2.832
Lachnospiraceae	3.00×10^{-2}	5.320[a]	2.174	5.319[a]	2.687	14.092[b]	7.738
Rikenellaceae	1.86×10^{-3}	2.759[a]	1.531	12.945[b]	8.865	15.281[b]	10.019
vadinBB60 group	4.23×10^{-5}	65.893[a]	8.715	48.119[b]	22.646	19.253[c]	14.792
**At the genus level**							
vadinBB60_unclassified	6.23×10^{-5}	65.893[a]	8.715	48.119[b]	22.646	19.253[c]	14.792
RC9-gut group	7.30×10^{-3}	1.228[a]	1.393	10.885[b]	9.036	12.367[b]	9.792
Desulfovibrio	1.39×10^{-2}	4.485[a]	1.703	9.915[b]	5.906	9.084[b]	2.781
Allobaculum	3.40×10^{-2}	3.019[a]	2.723	3.328[a]	4.695	11.103[b]	20.940
Blautia	4.10×10^{-2}	1.215[a]	0.969	1.784[a]	1.540	5.219[b]	2.565
Bacteroides	3.10×10^{-2}	0.109[a]	0.080	0.164[a]	0.130	0.543[b]	0.573
Clostridium	4.50×10^{-2}	0.163[a]	0.165	0.262[a]	0.156	0.583[b]	0.683
Roseburia	9.30×10^{-3}	0.040[a]	0.047	0.058[a]	0.080	0.686[b]	0.484
Lactobacillus	6.00×10^{-3}	0.006[a]	0.011	0.020[a]	0.039	0.306[b]	0.334

Statistical analysis was performed using Benjamini-Hochberg false discovery rate. Superscript letters assignation for each bacterial taxa reflecting paired statistical difference ($p < 0.05$) according to two-way ANOVA, followed by the Tukey post hoc test. SD: standard deviation.
